# Complications after hip arthroplasty and the association with hospital procedure volume: A nationwide retrospective cohort study on 50,080 total hip replacements with a follow-up of 3 months after surgery

**DOI:** 10.3109/17453674.2011.618907

**Published:** 2011-11-24

**Authors:** Laura M de Vries, Miriam CJM Sturkenboom, Jan AN Verhaar, J Herre Kingma, Bruno HCh Stricker

**Affiliations:** ^1^Dutch Health Care Inspectorate, the Hague; ^2^Pharmaco-Epidemiology Unit, Departments of Epidemiology and Medical Informatics, Erasmus Medical Center; ^3^Department of Orthopedics, Erasmus Medical Center, Rotterdam; ^4^Hospital Medisch Spectrum Twente, Enschede, the Netherlands

## Abstract

**Background and purpose:**

It has been suggested that a higher procedure volume is associated with less complications after hip arthroplasty. In order to investigate the incidence of serious negative outcomes and a possible association with procedure volume, we performed a retrospective nationwide cohort study on total hip replacements in all Dutch hospitals.

**Methods:**

All total hip replacements (n = 50,080) that were identified as primary intervention in all general and university medical centers between January 1, 2002 and October 1, 2004 were included. Primary endpoints of follow-up were mortality and complications during admission, and re-admission within 3 months due to complications. Variables that were assessed as potential risk factor were age, sex, duration of (preoperative) admission, specific diagnosis, acute/non-planned admission, co-morbidity, and hospital procedure volume.

**Results:**

Age, sex, and comorbidity were associated with complications and mortality. Additionally, acute admission was a risk factor for mortality but not for complications. There was no linear trend indicating that decreasing volume led to an increasing number of complications, and no statistically sginificant effect for mortality was found.

**Interpretation:**

After adjustment for several risk factors, we found that the hospitals performing most hip procedures every year had fewer complications during index admission, but that they did not have a lower mortality than groups performing fewer procedures. The lack of a linear trend may be explained by the fact that almost all Dutch hospitals perform a high number of hip arthroplasties each year.

Approximately 20,000 total hip replacements are performed in Dutch general and university hospitals each year (Prismant 2009). It is expected that this number will increase to more than 30,000 in 2030 and to more than 50,000 in the longer term (Otten et al. 2010). Mortality, significant blood loss, postoperative infections, deep venous thrombosis (DVT), dislocations of the prosthesis, and instability are the most common early complications. Risk factors for complications are the type of intervention (hemiarthroplasty, total hip replacement, revision, trauma surgery), age, sex, and other patient-related factors such as obesity (Lübbeke et al. 2007). Furthermore, several studies have shown an association between complications on the one hand and experience of the surgeon and the hospital on the other, expressed as annual number of hip arthroplasties (Kreder et al. 1997, Katz et al. 2001, Solomon et al. 2002, Losina et al. 2004, Battaglia et al. 2006, Doro et al. 2006, Judge et al. 2006, Cram et al. 2007, Shervin et al. 2007, Manley et al. 2008, Bozic et al. 2010, SooHoo et al. 2010).

Most studies have been performed in the United States, and due to differences in healthcare systems, it is not clear whether these results can be generalized to other countries. The aim of our retrospective nationwide cohort study was to gain insight into the incidence and risk of several serious complications of hip arthroplasty, both during the index hospitalization period and within the first 3 months after surgery. In addition, we assessed the importance of risk factors for complications such as the experience of the hospital, expressed as the number of interventions performed annually and corrected for several patient-related factors such as age, sex, co-morbidity, and diagnosis.

## Patients and methods

### Setting

Data were retrieved from a nationwide computer database of hospital discharge records, with complete coverage of all admissions in all general and university hospitals in the Netherlands (which has 16 million inhabitants). None of these hospitals is private. The university hospitals are owned by the government and the general hospitals are independent foundations, financed by public money. Private clinics did not perform THAs. The database includes (among other information) basic patient characteristics, date of admission and discharge, the main intervention (coded), the medical specialist (coded), and the main and secondary diagnoses at discharge, based on the ICD-9-CM coding system (ICD-9-CM, 1978). Characteristics of hospitalizations are registered by treating medical specialists or residents and coded by professional code clerks on the basis of hospital discharge letters. For every admission, one main diagnosis or diagnosis at discharge (mandatory) and up to 9 secondary diagnoses (optional) are registered. The coding is independent of reimbursement of the hospital or specialist. In addition, hospitals remain anonymous with the use of unique codes, instead of name and address data. All diagnoses are submitted in the same format, mostly electronically.

### Cohort and outcome definition

All patients admitted for a first total hip arthroplasty between January 1, 2002 and October 1, 2004 were included in this nationwide cohort study (n = 50,080). This was the most recent dataset available, with sufficient power due to the large number of records. Each cohort member was followed only once from the day of the hip arthroplasty (index hospitalization) until the earliest of one of the following events: death during index admission, a complication, or end of the follow-up time of 3 months, whichever came first. Patients with an ICD-9 code indicating certain non-fatal complications related to the implant, such as mechanical loosening, dislocation, or infection of the implant during the index hospitalization were excluded since these complications may have been related to an earlier intervention and not to the index intervention that was performed during the study period. All interventions with codes indicating removal or revision of hip implants were excluded from the database, except when such removals or revisions occurred within 3 months after the index operation, as they may have indicated a complication.

In the total population, we identified 82,582 admissions for hemiarthroplasty and total hip arthroplasty in the period 2002–2004. After exclusion of patients who were admitted in 2001 but discharged in 2002, admissions later than September 30, 2004 to ensure at least 3 months of follow-up, patients discharged with codes indicating a complication from an earlier procedure, and patients with certain types of malignancies, fractures, and hemiarthroplasties, the study cohort consisted of 50,080 admissions for primary total hip arthroplasty.

In the Netherlands, most patients with a hip fracture have a hemiarthroplasty procedure, while patients with osteoarthrosis receive a total hip replacement. Patients with a fracture are clinically different from patients with osteoarthrosis. Thus, we excluded patients with fractures from the study cohort. Additionally, patients with osteoarthrosis or another diagnosis that was not fracture were excluded if they had hemiarthroplasty (see Figure).

**Figure:**
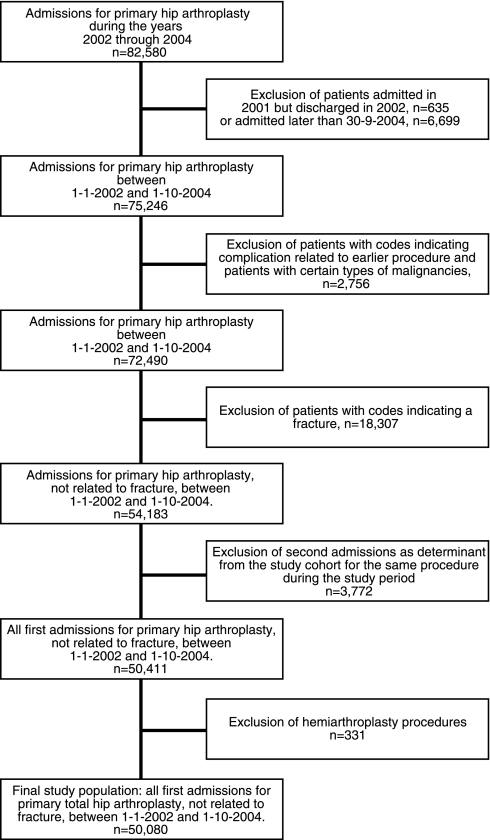
Schematic representation of exclusion of patients from the study population.

We assessed the proportion of deaths and complications that occurred during the index hospitalization and the proportion of re-admissions as a result of a selected set of potential complications within 3 months of the index hospitalization. We searched the original database records over the period of April 1, 2002 to January 1, 2005 for admitted patients with the same date of birth, sex, postal code of their home address, and hospital identification code, as we found that almost all patients were re-admitted to the same hospital. Complications were identified based on the ICD-9 codes of the main discharge diagnoses and on literature (Kreder et al. 1997, Katz et al. 2001). As complications during index hospitalization we considered ICD-9 codes describing pulmonary embolism, specific complications affecting specified body systems such as cardiac, vascular, respiratory, or urinary complications, complications of procedures, and complications of medical care. Reasons for re-admission that were considered as complications were: several types of infection, dislocation, vascular complications, and complications due to the procedure or implant. For this, we used a specific list, which was described earlier by Kreder et al. (1997) and Katz et al. (2001). In addition, removal or revision of hip arthroplasty within 3 months of the index operation was also considered to be an indication of a complication and was therefore included as an endpoint.

### Hospital volume

During the study period, the Netherlands had 88 general hospitals and 8 university medical centers. 2 of the 96 hospitals only performed hemiarthroplasties; therefore, 94 hospitals remained in the study. Some hospitals have more than one location, but for the purpose of our research they were considered as one organization. The Netherlands is a densely populated country with a relatively old population. The average number of hip procedures per hospital may therefore be higher than in other countries. Only 13 hospitals performed less than 100 total hip arthroplasties annually during the study period. When hemiarthroplasties and fractures were also considered, this number was even lower.

We divided the hospitals into 5 volume groups based on the mean number of total hip arthroplasties performed per year. The lowest volume group performed less than 100 procedures a year and the highest volume group performed more than 400 procedures a year. The number of patients in each of these groups (see Tables 3 and 4) is the number of patients from the total cohort who were in that group during the study period of 2.75 years.

Our data did not allow us to distinguish between individual surgeons. Orthopedic surgeons performed almost all of the total hip replacements (99.9%).

### Covariables

As covariables, the following variables were considered for inclusion in the models: age and sex, surgical procedure volume per hospital, and co-morbidity in the year prior to the intervention that was severe enough for hospitalization and diagnosis. In order to assess co-morbidity a year before surgery, we searched the original database records over the period January 1, 2001 to January 1, 2005 for admitted patients who had the same date of birth, sex, and postal code. We classified co-morbidity according to the Charlson co-morbidity index (Charlson et al. 1987) as adapted by Deyo et al. for ICD-9 databases (Deyo et al. 1992). Furthermore, we considered the diagnosis, whether admission was acute and unplanned, duration of admission before surgery and total duration of admission.

### Validation

We performed validation of procedures, complications, and mortality in a sample of our study material by linking it to the Rotterdam Study, a prospective population-based cohort study of chronic diseases in the elderly who live in the Ommoord district of the city of Rotterdam (Hofman et al. 1991, 2007, 2009).

By matching according to date of birth, sex, and postal code of the home address, we identified 68 patients from the study cohort in the Rotterdam Study. These 68 patients had been admitted to 3 hospitals in Rotterdam and surrounding area. For 40 patients, the original file including the original discharge letter was available for review. For 17 other patients, information from their general practitioner could be accessed digitally, and no information was available for the other 11 patients.

Of all the procedures, diagnoses, and complications, 91% (CI: 84–99), 90% (CI: 82–97), and 80% (CI: 45–115), respectively, were confirmed. The remainder was missing and could not be judged. However, no procedures, diagnoses, or complications were false-positive.

### Analysis

Descriptive analyses were conducted using SPSS software version 15.0. Statistical comparison of means and proportions consisted of independent samples t-tests (Student's), and chi-square tests. Because the precise delay between hip arthroplasty and complications during the index hospitalization was not available in the database, we used logistic regression analysis with the first occurrence of a complication as endpoint instead of a Cox proportional hazards model. Covariables that were considered as risk factors in the literature were tested in a univariable logistic regression analysis in order to obtain crude odds ratios. The final multivariable models were fitted by backward elimination regression based on the maximum likelihood ratio (Sun et al. 1996). The effect of a variable on the risk of complication was expressed as an odds ratio with a 95% confidence interval (CI).

## Results

Approximately half of the patients were older than 70 years, with a mean age of 69 years (SD 11), and about 70% were women. The median duration of admission was 9 days, with a median preoperative stay of 1 day. Most patients were admitted with a diagnosis of osteoarthritis (97%) and 7% had one or more co-morbidities. About 1% (n = 504) of the admissions were not planned. Most of these patients (n = 408, 81%) had osteoarthritis as the main diagnosis, 9.5% (n = 48) had other bone defects such as aseptic bone necrosis and malunion/nonunion of fracture, and another 9.5% (n = 48) had a variety of other diagnoses; 11 of the patients of this group had osteoarthritis as secondary diagnosis. The reason for these admissions being acute/unplanned was not mentioned. The mortality rate during the index admission was 0.2% (n = 114), and 2.2% (n = 1,115) of the patients had one or more complications. Including re-admissions during the 3 months after surgery, 5.8% (n = 2,880) of the patients had a complication—either during the index admission or after re-admission for that reason (Table 1).

**Table 1.** Baseline characteristics of the study cohort (n = 50,080)

Characteristics: Primary total hip replacement	All admissions	Males	Females
	n = 50,080	n = 14,966, 30%	n = 35,114, 70%
Mean age, years (SD)	68.7 (10.6)	65.9 (11.0)	70.0 (10.1)
Median age, years (range)	70 (15–99)	67 (15–99)	71 (15–99)
Median duration of admission, days (range)	9 (1–137)	8 (1–137)	9 (1–133)
Median duration of preoperative admission, days (range)	1 (0–80)	1 (0–80)	1 (0–76)
Acute, unplanned admission, n (%)	504 (1.0)	132 (0.9)	372 (1.1)
Any co-morbidity, n (%)	3,423 (6.8)	1,164 (7.8)	2,259 (6.4)
Specialist:			
Orthopedic surgeon, n (%)	50,038 (99.9)	14,956 (99.9)	35,082 (99.9)
General surgeon, n (%)	34 (0.1)	9 (0.1)	25 (0.1)
Other surgeon, n (%)	8 (< 0.1)	1 (< 0.1)	7 (< 0.1)
Died during admission (all causes), n (%)	114 (0.2)	42 (0.3)	72 (0.2)
Complication during admission, n (%)	1,115 (2.2)	350 (2.3)	765 (2.2)
Re-admitted with a complication at least once within 3 months	1,765 (3.5)	595 (4.0)	1,170 (3.3)
Any unfavorable outcome **^a^**	2,880 (5.8)	947 (6.3)	1,933 (5.5)
Diagnosis			
Osteoarthritis, n (%)	48,313 (96.5)	14,260 (95.3)	34,053 (97.0)
Aseptic bone necrosis, n (%)	937 (1.9)	419 (2.8)	518 (1.5)
Congenital deformity of hip, n (%)	141 (0.3)	37 (0.2)	104 (0.3)
Rheumatoid arthritis, n (%)	121 (0.2)	31 (0.2)	90 (0.3)
Other, n (%)	568 (1.1)	219 (1.5)	349 (1.0)

**^a^** Complication during index admission and/or re-admission due to complication up to 3 times within 3 months of surgery.

Almost 9% (n = 4,364) of the patients were re-admitted at least once within 3 months of surgery. About 40% (n = 1,756) were re-admitted with a complication of the procedure, most of which involved a mechanical complication of the device (51%, n = 892) or an infection (30%, n = 526). The second and third re-admissions within the same time frame showed a similar picture (Table 2). Of these 4,364 patients, about 10% were admitted for a second hip replacement.

**Table 2.** Characteristics of first 3 re-admissions within 3 months of surgery

	First		Second		Third	
	n = 4,364		n = 759		n = 167	
	n	% (95% CI)	n	% (95% CI)	n	% (95% CI)
Second total hip replacement	423	9.7 (8.8–10.6)	25	3.3 (2.0–4.6)	3	1.8 (–0.2 to 3.8)
Complication during index admission	156	3.6 (3.0–4.1)	34	4.5 (3.0–6.0)	6	3.6 (0.8–6.4)
Re-admission was acute/unplanned	2,534	58.1 (56.6–59.5)	397	52.3 (48.8–55.9)	72	43.1 (35.6–50.6)
Mortality during re-admission	83	1.9 (1.5–2.3)	9	1.2 (0.4–2.0)	5	3.0 (0.4–5.6)
Readmission was due to complication	1,765	40.4 (39.0–41.9)	322	42.4 (38.9–46.0)	70	41.9 (34.4–49.4)
Complications specified:	1,765	100	322	100	70	100
Mechanical complication of device	892	50.5 (48.2–52.9)	212	65.8 (60.7–71.0)	43	61.4 (50.0–72.8)
Infection	526	29.8 (27.7–31.9)	73	22.7 (18.1–27.2)	20	28.6 (18.0–39.2)
Dislocation	47	2.7 (1.9–3.4)	10	3.1 (1.2–5.0)	0	0.0 (0.0–0.0)
Pulmonary embolism	58	3.3 (2.5–4.1)	5	1.6 (0.2–2.9)	1	1.4 (–1.4 to 4.2)
Deep vein thrombosis	61	3.5 (2.6–4.3)	4	1.2 (0.03–2.5)	1	1.4 (–1.4 to 4.2)
Other	180	10.2 (8.8–11.6)	18	5.6 (3.1–8.1)	5	7.1 (1.1–13.2)

Table 3 shows the univariable and multivariable analyses of all risk factors associated with mortality during the index admission. Age, male sex, co-morbidity, and certain diagnoses appeared to be associated with mortality. Furthermore, a complication during the index admission was a risk factor for mortality, with an adjusted odds ratio of 13 (CI: 8–21). Hospital groups performing fewer procedures appeared to be associated with a lower risk of mortality than the hospital group that performed most interventions. However, the odds ratios did not reach statistical significance.

**Table 3.** Adjusted associations of all risk factors with mortality per hospital group

	Early mortality during index admission	
	Crude OR (95% CI)	Adjusted OR (95% CI)
Hospital volume as no. of total hip procedures **^a^** per year		
> 400 (n_h_ = 7 / n_p _= 8,813) **^b^**	ref	ref
300–400 (n_h_ = 12 / n_p_ = 10,260)	0.94 (0.52–1.7)	0.8 (0.44–1.5)
200–300 (n_h_ = 20 / n_p_ = 12,413)	1.2 (0.67–2.0)	1.1 (0.6–2)
100–200 (n_h_ = 42 / n_p_ = 16,196)	0.91 (0.53–1.6)	0.76 (0.44–1.3)
< 100 (n_h_ = 13 / n_p_ = 2,398)	0.18 (0.02–1.3)	0.17 (0.02–1.2)
Female sex	0.73 (0.5–1.1)	0.58 (0.39–0.86)
Age (quartiles of no. of patients)		
≤ 65	ref	ref
66–72	1.8 (0.81–4.0)	2 (0.88–4.4)
73–79	5.6 (2.8–11)	5.8 (2.9–12)
≥ 80	11 (5.5–22)	11 (5.4–22)
Co–morbidity **^c^**		
Score 0	ref	ref
Score 1	3.9 (2.3–6.4)	2.7 (1.6–4.6)
Score 2	6.8 (3.1–15)	4.2 (1.9–9.3)
Score 3	12 (4.7–29)	6 (2.3–16)
Diagnosis		
Osteoarthritis	ref	ref
Aseptic bone necrosis	3.3 (1.5–7.6)	3.2 (1.4–7.6)
Congenital deformity of hip	–	–
Rheumatoid arthritis	4.3 (0.59–31)	2.3 (0.29–18)
Other	12 (6.7–22)	7.9 (4.1–15)
Acute, unplanned admission	6.5 (3–14)	3 (1.2–7.2)
Duration of index admission	1 (1–1.1) **^d^**	1 (0.95–1) **^e^**
Duration of preoperative admisison	1.1 (1–1.1) **^f^**	– **^g^**
Complication during index admission	18 (12–27)	13 (8.4–21)

**^a^** Fractures and hemiarthroplasties excluded.

**^b^** n_h _= no. of hospitals / n_p_ = no. of patients in group.

**^c^** Charlson co-morbidity index, adapted by Deyo et al. for ICD–9 databases

**^d^** Results with 2 decimals: 1.04 (1.02–1.05).

**^e^** Results with 3 decimals: 0.972 (0.947–0.999).

**^f^** Results with 2 decimals: 1.06 (1.03–1.10).

**^g^** Not in final multivariable model.

Table 4 shows the univariable and multivariable analysis of several risk factors with complications during the index admission and the first 3 re-admissions within 3 months of surgery.

**Table 4.** Crude and adjusted associations of all risk factors with complications during index admission and re–admission

	Complication during index admission		Re-admission due to complication	
	Crude OR (95% CI)	Adjusted OR (95% CI)	Crude OR (95% CI)	Adjusted OR (95% CI)
Hospital volume as no. of total hip procedures **^a^** per year				
> 400 (n_h_ = 7 / n_p_ = 8,813) **^b^**	ref	ref	ref	ref
300–400 (n_h_ = 12 / n_p _= 10,260)	2.2 (1.7–2.8)	2.2 (1.7–2.7)	1.3 (1.1–1.5)	1.3 (1.1–1.5)
200–300 (n_h_ = 20 / n_p_ = 12,413)	2 (1.6–2.5)	1.9 (1.5–2.4)	1 (0.89–1.2) **^c^**	1 (0.88–1.2) **^d^**
100–200 (n_h_ = 42 / n_p_ = 16,196)	2 (1.6–2.5)	1.8 (1.5–2.3)	0.96 (0.84–1.1)	0.94 (0.82–1.1)
< 100 (n_h_ = 13 / n_p_ = 2,398)	2.3 (1.7–3.2)	1.9 (1.4–2.6)	1.1 (0.9–1.4)	1.1 (0.87–1.4)
Female sex	0.93 (0.82–1.1)	0.78 (0.68–0.9)	0.83 (0.75–0.92)	0.79 (0.71–0.87)
Age (quartiles of no. of patients)				
≤ 65	ref	ref	ref	ref
66–72	1.3 (1.1–1.6)	1.2 (1–1.5) **^e^**	1.1 (0.93–1.2)	1.1 (0.98–1.3)
73–79	2 (1.7–2.4)	1.5 (1.3–1.8)	1.5 (1.3–1.7)	1.6 (1.4–1.8)
≥ 80	2.6 (2.2–3.2)	1.5 (1.3–1.9)	1.7 (1.5–2)	1.9 (1.6–2.2)
Co–morbidity **^f^**				
Score 0	ref	ref	ref	ref
Score 1	2.1 (1.7–2.5)	1.7 (1.3–2.1)	1.5 (1.3–1.8)	1.4 (1.2–1.7)
Score 2	2.5 (1.7–3.7)	1.8 (1.2–2.7)	1.7 (1.2–2.4)	1.5 (1.1–2.2)
Score 3	3.1 (1.9–5.3)	1.9 (1–3.4) **^g^**	2 (1.2–3.3)	1.8 (1.1–2)
Diagnosis				
Osteoarthritis	ref	ref	ref	ref
Aseptic bone necrosis	2.1 (1.5–2.9)	1.8 (1.3–2.6)	1.8 (1.4–2.3)	1.8 (1.4–2.4)
Congenital deformity of hip	–	–	0.19 (0.03–1.4)	0.22 (0.03–1.6)
Rheumatoid arthritis	1.2 (0.37–3.7)	0.6 (0.16–2.2)	3 (1.6–5.4)	2.6 (1.4–4.8)
Other	3.9 (2.8–5.3)	2.5 (1.7–3.5)	1.8 (1.2–2.5)	1.8 (1.2–2.5)
Acute, unplanned admission at index admission	1.8 (1.2–2.9)	– **^h^**	0.81 (0.49–1.4)	– **^h^**
Duration of index admission	1.1 (1.1–1.1) **^i^**	1.1 (1.1–1.1) **^j^**	1 (1–1) **^k^**	1 (0.98–1) **^l^**
Duration of preoperative admission	1 (1–1.1) **^m^**	0.87 (0.84–0.91)	0.99 (0.96–1)	– **^h^**
Complication during index admission	NA	NA	2 (1.6–2.5)	1.9 (1.5–2.5)

**^a^** Fractures and hemiarthroplasties excluded.

**^b^** n_h _= no. of hospitals / n = no. of patients in that group.

**^c^** Results with 2 decimals: 1.04 (0.89–1.20).

**^d^** Results with 2 decimals: 1.02 (0.88–1.19).

**^e^** Results with 2 decimals: 1.22 (1.01–1.46).

**^f^** Charlson co-morbidity index, adapted by Deyo et al. for ICD–9 databases.

**^g^** Results with 2 decimals: 1.89 (1.04–3.41).

**^h^** Not in final multivariable model.

**^i^** Results with 2 decimals: 1.10 (1.09–1.10).

**^j^** Results with 3 decimals: 1.103 (1.095–1.110).

**^k^** Results with 3 decimals: 1.004 (0.996–1.012).

**^l^** Results with 3 decimals: 0.986 (0.977–0.995).

**^m^** Results with 2 decimals: 1.04 (1.02–1.06).

Age, male sex, co-morbidity, and diagnosis (aseptic bone necrosis and other) were statistically significantly associated with both endpoints. Hospital volume appeared to be associated with complications during the index admission, as all lower-volume groups had higher odds ratios than the high-volume group. However, it did not show the linear trend that would have been expected. For re-admissions due to complications, this association was not apparent.

## Discussion

In this study we found that during the index admission for total hip replacement, the percentage of complications was 2.2%. Almost 9% of the patients in the study cohort who were admitted between January 1, 2002 and October 1, 2004 for THA were re-admitted for any cause at least once within 3 months of surgery. However, 40% of these re-admissions were due to a complication that could be related to the implantation. Altogether, approximately 6% of the cohort of patients studied experienced a complication during index admission and/or within 3 months after the implantation. Acute admission appeared to be a risk factor for complications during index admission in the unadjusted analysis, but it was not selected as a risk factor in the final model. This may have been caused by adjustment for co-morbidity and diagnosis, variables that may confound the effect of acute admission.

The mortality during admission was 0.2% for patients with a total hip replacement. A complication during index hospitalization was strongly associated with mortality. However, as might be expected, mortality could not be entirely related to the intervention, since age, co-morbidity, and acute admission (i.e. trauma) were also associated with it. The high-volume group had a higher risk of mortality than 3 of the lower-volume groups. This may be explained by the fact that complicated total hip replacements are usually referred to high-volume centers. However, we did not find that higher hospital volume was associated with lower mortality, as found in previous studies (Shervin et al. 2007, Bozic et al. 2010). Furthermore, we must note here that the data came from the Dutch National Medical Registration, which records all hospital admissions until discharge. Thus, only mortality during admission is registered in this database, and we were unable to monitor mortality after discharge. SooHoo et al. (2010) found a mortality rate of 0.7% and a complication rate of 3.8% within 90 days. It is possible that the mortality rate is higher and shows more differences between volume groups when mortality that occurs after discharge is taken into account. Mortality within 3 months of surgery may still be related to the procedure, although the rate is rather low due to modern advances in surgery, anesthesia, and rehabilitation—and despite early discharges, operations on older and more fragile individuals, and earlier rehabilitation (Pulido et al. 2008, Aynardi et al. 2009).

In the past decade, several studies have been performed on the incidence of complications following surgery and the effect of hospital and surgical procedure volume (Kreder et al. 1997, Katz et al. 2001, Losina et al. 2004, Battaglia et al. 2006, Doro et al. 2006, Judge et al. 2006, Cram et al. 2007, Shervin et al. 2007, Manley et al. 2008, Bozic et al. 2010). Many of these studies had a follow-up time of 3 months after surgery, since it appeared that the largest proportion of complications manifests itself within that time period, an extensive proportion of which occurred within a few days of surgery (Kreder et al. 1997, Katz et al. 2001, Philips et al. 2003, Parvizi et al. 2007). Primary endpoints were mortality, infection, dislocation and/or instability, deep vein thrombosis, and pulmonary embolism. In our study, we investigated the occurrence of any complication, including the above-mentioned outcomes, and infection, dislocation, deep vein thrombosis, and pulmonary embolism separately.

Although hospital groups performing a lower number of THRs were more strongly associated with complications during the index admission than the highest-volume group, our study did not show a trend towards a lower proportion of complications when the number of interventions per hospital increased. Furthermore, there was no association between volume groups and re-admissions within 3 months. As the average number of hip arthroplasties per hospital was high in our study, this may have removed the potential difference between high-volume and low-volume hospitals. However, as in another study that did not find an effect of hospital volume on outcome (Zahn et al. 2007), and according to privacy legislation, our administrative data did not allow us to identify the surgeons who performed the intervention. Several other studies showed that a higher volume of hip arthroplasties resulted in a lower incidence of complications, and suggested that procedure volume per surgeon is the most important determinant (Lavernia and Guzman 1995, Losina et al. 2004, Battaglia et al. 2006, Shervin et al. 2007, Manley et al. 2008). This was questioned in other studies, where the authors concluded that hospital volume is an important factor (Doro et al. 2006). It was also found that in patients who were operated by higher-volume surgeons, higher hospital volume was independently associated with lower early failure rates (Losina et al. 2004), but specialization of the hospital may also play a role (Cram et al. 2007). Furthermore, it has been suggested that the volume effect reaches a plateau and does not improve further regardless of increasing volume (Sharkey et al. 2004). Thus, hospital volume may still be an appropriate indicator of quality (Meredith and Katz 2007).

Finally, there may have been residual confounding caused by factors that could not be adjusted for in our analysis, such as type of prosthesis used, facilities in the operating room, and so on.

As far as we know, our study is the first nationwide study in which all primary total hip replacements have been included, and involves a high average number of hip procedures per hospital in a densely populated country. Also, validation of a sample showed that the quality of the registry is good. Furthermore, it is unlikely that there was selection bias, since Dutch inhabitants in need of hip arthroplasty are generally admitted to a Dutch hospital. Information bias due to knowledge of the research question was also unlikely, as the admissions were registered prospectively. However, false-negative misclassification of cases could be an issue because we only had access to data about mortality during hospitalization. Although a median length of stay of 9 days is rather long for this type of procedure, it is still likely that some mortality related to the procedure occurred after discharge. Additionally, although trained code clerks register hospital admissions, mistakes in coding and deviations between employees and hospitals cannot be excluded. However, given the high concordance between registered determinants and complications and medical records in our validation sample, we think that this is unlikely.

At least one additional diagnosis was mentioned in only 25% of the admissions. Possible complications may not have been registered because such registration is optional and only registration of the main diagnosis is mandatory. This may have led to underestimation of the number of complications.

In conclusion, in contrast with results from USA hospitals, volume does not fully explain the differences in mortality and complications between hospitals in the Netherlands. This might be explained in part by the fact that the average number of hip arthroplasties per hospital is high. This might mean that under such circumstances, other determinants become more important in explaining differences—such as volume per surgeon or technical considerations such as type of prosthesis, surgical technique, use of cement, conditions in the operating rooms, or patient related factors. Other options that may be interesting for further investigation are (1) the degree of orthopedic specialization of hospitals, as an association between this parameter and favorable outcome has been shown in a recent study (Hagen et al. 2010), and (2) the importance of standardization of the process, which was found to be strongly associated with better patient outcomes and more efficient use of resources in another study (Bozic et al. 2010).
